# Explicit Instructions Do Not Enhance Auditory Statistical Learning in Children With Developmental Language Disorder: Evidence From Event-Related Potentials

**DOI:** 10.3389/fpsyg.2022.905762

**Published:** 2022-06-30

**Authors:** Ana Paula Soares, Francisco-Javier Gutiérrez-Domínguez, Helena M. Oliveira, Alexandrina Lages, Natália Guerra, Ana Rita Pereira, David Tomé, Marisa Lousada

**Affiliations:** ^1^Human Cognition Lab, CIPsi, School of Psychology, University of Minho, Braga, Portugal; ^2^Psychological Neuroscience Lab, CIPsi, School of Psychology, University of Minho, Braga, Portugal; ^3^Department of Audiology, School of Health, Polytechnic Institute of Porto, Porto, Portugal; ^4^Neurocognition Group, Laboratory of Psychosocial Rehabilitation, CiR, Porto, Portugal; ^5^Center for Health Technology and Services Research (CINTESIS@RISE), School of Health Sciences, University of Aveiro, Aveiro, Portugal

**Keywords:** developmental language disorder, statistical learning, implicit learning, explicit learning, SL deficit hypothesis, procedural deficit hypothesis, word predictability, ERP word segmentation correlates

## Abstract

A current issue in psycholinguistic research is whether the language difficulties exhibited by children with developmental language disorder [DLD, previously labeled specific language impairment (SLI)] are due to deficits in their abilities to pick up patterns in the sensory environment, an ability known as statistical learning (SL), and the extent to which explicit learning mechanisms can be used to compensate for those deficits. Studies designed to test the compensatory role of explicit learning mechanisms in children with DLD are, however, scarce, and the few conducted so far have led to inconsistent results. This work aimed to provide new insights into the role that explicit learning mechanisms might play on implicit learning deficits in children with DLD by resorting to a new approach. This approach involved not only the collection of event-related potentials (ERPs), while preschool children with DLD [relative to typical language developmental (TLD) controls] were exposed to a continuous auditory stream made of the repetition of three-syllable nonsense words but, importantly, the collection of ERPs when the same children performed analogous versions of the same auditory SL task first under incidental (implicit) and afterward under intentional (explicit) conditions. In each of these tasks, the level of predictability of the three-syllable nonsense words embedded in the speech streams was also manipulated (high vs. low) to mimic natural languages closely. At the end of both tasks’ exposure phase, children performed a two-alternative forced-choice (2-AFC) task from which behavioral evidence of SL was obtained. Results from the 2-AFC tasks failed to show reliable signs of SL in both groups of children. The ERPs data showed, however, significant modulations in the N100 and N400 components, taken as neural signatures of word segmentation in the brain, even though a detailed analysis of the neural responses revealed that only children from the TLD group seem to have taken advantage of the previous knowledge to enhance SL functioning. These results suggest that children with DLD showed deficits both in implicit and explicit learning mechanisms, casting doubts on the efficiency of the interventions relying on explicit instructions to help children with DLD to overcome their language difficulties.

## Introduction

Learning to talk is one of the most astonishing abilities children achieve during infancy. Indeed, within a few years, they go from cooing and babbling to an extraordinary complex use of the sounds of the language spoken around them to communicate their needs, feelings, and thoughts. Although most children acquire this remarkable ability quickly, effortlessly, and with no need for any explicit instructions, a nonnegligible portion (~7%–10%) shows significant problems in using speech and language to communicate (e.g., Tomblin et al., 1997; Norbury et al., 2016), presenting a development language disorder (DLD).

The term DLD was introduced by Bishop et al. (2017) to refer to children who showed significant language difficulties at expressive and/or receptive levels, impacting their daily lives and/or their educational outcomes not only during infancy but also typically throughout their entire lives (see Conti-Ramsden et al., 2018). DLD occurs in the absence of other neurodevelopmental disorders, such as autism spectrum disorder (ASD) or intellectual disability (ID), brain injury, hearing loss, or a known biomedical condition (e.g., genetic conditions). The term was introduced to replace the specific language impairment (SLI; Leonard, 1981, 2014) label, widely used in research since the mid-1980s (see, however, Bishop, 2014 for a review of other terms in clinical and educational contexts), because the strict use of the term SLI excludes from diagnosis a significant number of children who struggle with relevant language difficulties, which might pose a greater challenge for them to have access to specialized health services that could mitigate the detrimental effects this condition brings to these children, their families, and the society as a whole (see Bishop et al., 2016, 2017 and Soares et al., 2021b for details). The definition of DLD is thus broader than SLI since it also includes children whose language problems may co-occur with other motor, cognitive, emotional, and/or behavioral disorders, such as attention-deficit hyperactivity disorder (ADHD), developmental coordination disorder (DCD), or developmental dyslexia (DD). It also includes children scoring one SD below the mean in nonverbal intelligence quotient (NVIQ) standardized scales (i.e., NVIQ < 85) that were excluded from the SLI diagnosis, even though those scoring within the range that qualifies them for intellectual disability (i.e., two SDs below the mean or scores of NVIQ < 70) is still excluded from the DLD diagnosis. This change in the diagnostic criteria responds to compelling evidence, showing that children with selective language impairments (i.e., those who meet the SLI criteria) are relatively rare, and there is no evidence that they respond differently to intervention, or that they present a different psycholinguistic profile than children with language difficulties that do not completely meet SLI criteria (e.g., Dyck et al., 2011; Reilly et al., 2014; Norbury et al., 2016; Lancaster and Camarata, 2019; McGregor et al., 2020).

The etiology of DLD is complex and hotly debated in the current research, with approaches claiming that the language difficulties observed by these children arise from impairments that are specific to grammar (e.g., late parameterization, missing grammatical features, and representational deficits for dependent relationships, e.g., Rice and Wexler, 1996; Clahsen and Hansen, 1997; Leonard et al., 1997) to accounts arguing that the language impairments arise from deficits in the cognitive processes that subserve language but that are not specific to language (e.g., working memory, rapid temporal processing, and attention, e.g., Tallal et al., 1985; Archibald and Gathercole, 2006; Spaulding et al., 2008; Kidd, 2012). Here, we claim that the language difficulties observed in children with DLD might stem from deficits in their ability to extract patterns from the sensory environment without reinforcement or feedback, a cognitive ability known as statistical learning (SL)—see Perruchet and Pacton (2006) and Christiansen (2019) for other terms—and that is assumed to play a critical role in the acquisition of rule-governed aspects of language across phonology, morphology, and grammar (see Romberg and Saffran, 2010; Erickson and Thiessen, 2015; Saffran and Kirkham, 2018; Saffran, 2020). Indeed, in order to use language efficiently, children need to realize that despite the tremendous variability it presents at a surface level, language is a system governed by plenty of rules that define how speech sounds (phonemes) can be combined in the language to which they were exposed to generate words, how parts of words (morphemes) may be (re)arranged to create new words and to adjust them to the syntactic context in which they were used, and ultimately how words should be combined with each other to convey meaning (syntax).

Evidence for the involvement of SL mechanisms in language acquisition comes firstly from the seminal work of Saffran et al. (1996), showing that 8-month-old babies were able to compute the probability of a given syllable to be followed by another syllable in a continuous speech stream made of the repetition of four three-syllable nonsense words (e.g., “*gikobatokibutipolugopilatokibu*”), and to use that statistics, known as Transitional Probability (TP), to extract word-like units from the continuous speech (e.g., “*tokibu*,” “*gikoba*,” “*gopila*,” and “*tipolu*”). Note that, in that artificial language, TPs between syllables were higher within word boundaries (TP = 1.0) than across word boundaries (TP = 0.33), thus making the extraction of TPs a reliable cue for word segmentation. Since then, many other works have provided support for the involvement of SL mechanisms in other levels of language acquisition, such as word-referent associations (e.g., Saffran and Estes, 2006; Estes et al., 2007; Hay et al., 2011; Breen et al., 2019), grammatical categorization (e.g., Mintz, 2003), the establishment of long-distance dependencies in different grammatical structures (e.g., Gómez, 2002; Newport and Aslin, 2004; Gómez and Maye, 2005; Thompson and Newport, 2007; Kidd, 2012; Hsu et al., 2014), and literacy skills (e.g., Arciuli and Simpson, 2012; Spencer et al., 2015; Sawi and Rueckl, 2019; Lages et al., 2022). Statistical learning is, thus, assumed as a powerful mechanism that enables children to detect the regularities embedded in the spoken (and written) language even without awareness or intention to do so, and to use that “knowledge” to make predictions about “what comes next,” which not only facilitates language processing but also creates the conditions for children to scale up to the extraction of other (higher) levels of regularities that mastering a language requires.

Because extracting the patterns embedded in a language is assumed to be critical for language acquisition (see Romberg and Saffran, 2010; Erickson and Thiessen, 2015; Saffran and Kirkham, 2018; Saffran, 2020; Siegelman, 2020), and also because SL abilities vary considerably across individuals (e.g., Arciuli and Simpson, 2011; Misyak and Christiansen, 2012; Siegelman and Frost, 2015; Kidd and Arciuli, 2016; Johnson et al., 2020), it is not surprising that deficits in that ability had been put forward as a potential explanation for the difficulties exhibited by children with DLD (e.g., Evans et al., 2009, 2022; Lum et al., 2010, 2012, 2014; Hsu and Bishop, 2014; Arciuli and Conway, 2018; Plante and Gómez, 2018; Saffran, 2018; Soares et al., 2018; Ahufinger et al., 2021; Bogaerts et al., 2021). For instance, in a meta-analysis of studies using the serial reaction time task to test implicit learning in language-impaired participants, Lum et al. (2014) revealed that children with DLD performed poorly than typical language development (TLD) controls, even though the serial reaction time task contains an important motor learning component that seems also to be impaired in children with DLD (see Desmottes et al., 2016), which might have confounded the results. Nevertheless, in another meta-analysis targeting studies using a wide range of SL tasks in the visual and auditory domains (e.g., serial reaction time task and artificial grammar learning task), Obeid et al. (2016) found that children with DLD kept performing significantly below TLD controls, with task modality (visual vs. auditory) not moderating the effects, supporting the claim that SL is a general domain learning mechanism (see, however, Frost et al., 2015 for a discussion). Finally, Lammertink et al. (2017), in another meta-analysis focused on studies using word segmentation tasks (such as the triplet embedded task introduced by Saffran et al., 1996) and the artificial grammar learning task in the auditory domain using verbal materials, showed that children with DLD revealed significant impairments when compared with TLD controls in both tasks, leading the authors to conclude that the level of linguistic processing (word vs. grammar) did not modulate the results. Even though there are also studies showing that children with DLD performed just as well as TLD controls (see Lum and Bleses, 2012; Gabriel et al., 2014; Mayor-Dubois et al., 2016; West et al., 2018; Lammertink et al., 2020), the bulk of the studies conducted so far suggests that, on average, the extraction of the regularities embedded in the input seems to be impaired or, at least, not as effective in children with DLD as in peers controls (e.g., Tomblin et al., 1997; Evans et al., 2009, 2022), hence supporting the view of the existence of an SL deficit in DLD children.

The SL deficit hypothesis is also consistent with the procedural deficit hypothesis (Ullman and Pierpont, 2005; Ullman and Pullman, 2015; Ullman et al., 2020), stating that language difficulties observed in children with language impairments (e.g., DLD and DD) arise from a dysfunction in the procedural memory (PM) system. The PM system is a brain network connecting the cortex with the striatum in the basal ganglia (corticostriatal circuits), thought to be involved in implicit learning and to play a crucial role in the acquisition of the rule-governed aspects of language (e.g., morphosyntax and phonology). Additionally, the PDH also states that language difficulties observed in children with language impairments can be amended by the declarative memory (DM), a neural network located in the medial temporal lobe, thought to be largely spared (or even strengthened) in children with DLD and to assume a crucial role in the acquisition of the lexical-semantic aspects of language. Specifically, within that framework, it is claimed that children with DLD may store complex linguistic structures that normally are processed automatically in the PM system, such as decomposing morphological complex words into their constituents (e.g., “walked” → “walk” + “-ed”), in the DM system by the use of explicit rules (e.g., add “-ed” to a verb if the action has already occurred) or chunking, i.e., by storing these words as a whole (e.g., “walked”) in the mental lexicon. Evidence for the compensatory role of DM in children with DLD is, however, contentious. While some studies found intact or even enhanced performance in DM tasks in children with DLD relative to TLD controls, particularly in studies using DM tasks involving nonverbal materials (e.g., Riccio et al., 2007; Lum et al., 2010; Lum and Conti-Ramsden, 2013; Lukács et al., 2017; Earle and Ullman, 2021; see, however, Bishop and Hsu, 2015; Kuppuraj et al., 2016; Lee, 2018), others reported DM impairments, especially those using DM tasks involving verbal materials (e.g., Lum et al., 2010; Lukács et al., 2017; McGregor et al., 2017; Haebig et al., 2019; see, however, Baird et al., 2010; Evans et al., 2022), even though differences tend to vanish when working memory measures were taken into account (e.g., Alt and Plante, 2006; Lum et al., 2012, 2015; Arthur et al., 2021). Thus, it remains largely unknown whether children with DLD show or not deficits in the DM system and even if showing spared or enhanced DM performance, as some studies suggest, the extent to which these abilities can be effectively mobilized by DLD children to compensate for their PM deficits. Note that the studies conducted so far examining DM-PM functioning in children with DLD have relied on the use of different tasks and materials to test each of these functions (typically the serial reaction time task or the artificial grammar learning task to test PM functioning and face, object recognition, or word-lists tasks to test DM functioning) from a wide range of participants (children, adolescents, and adults), which could explain the disparity of the results. It is also important to emphasize that since these studies have relied on the collection of behavioral data (i.e., reaction time and accuracy measures), which can be strongly affected by attention and/or motivational factors, particularly those conducted with young participants, studies using other tasks and techniques, such as the Event-Related Potentials (ERPs) technique, are required to get a deeper understanding of the DM-PM dynamics in children with DLD with important theoretical and clinical implications.

## Current Study

The work presented here aimed to get new insights into the role that explicit learning mechanisms might play in implicit learning deficits in preschool children with DLD. For this, we resorted to a new approach that involved the collection of ERPs, while children with DLD (relative to TLD controls) were exposed to a continuous auditory stream made of the repetition of three-syllable nonsense words under two different conditions. First, they were exposed to a speech stream without any information regarding the task or the stimuli (i.e., under incidental conditions), and, subsequently, with previous knowledge about the regularities (word-like units) embedded in the input stream (i.e., under intentional conditions). The ERP technique is particularly well suited to study the compensatory role that explicit learning mechanisms might play on DLD since it allows to study the underpinnings of speech processing in the brain in young children with high time (millisecond) precision even in the absence of any overt response. These characteristics make ERPs an exceptional tool to overcome some of the problems that the exclusive use of behavioral measures (reaction times and/or accuracy) with young participants can bring to research (for details, see Daltrozzo and Conway, 2014; Royle and Courteau, 2014).

Following previous works (e.g., Soares et al., 2020, 2021a,b) in each auditory SL task (aSL), the level of predictability of the three-syllable nonsense words embedded in the speech streams was also manipulated (high vs. low) to mimic natural languages closely (see Soares et al., 2020 for details). At the end of the exposure phase of each aSL task, children performed a two-alternative forced-choice (2-AFC) task from which behavioral evidence of SL was obtained, as in most SL studies (for reviews, see Siegelman et al., 2017; Soares et al., 2022a). The collection of both neural and behavioral responses while preschool children, with and without DLD, performed analogous versions of the same task under implicit and explicit conditions. That allowed us to not only control for differences in the results that might have arisen in previous studies, from the use of different tasks and stimuli to test DM and PM functioning but also, importantly, to directly examine the changes that performing analogous versions of the same task, presented under different learning conditions, produced in the SL functioning. Note that although we recognize that the terms “declarative vs. procedural,” “explicit vs. implicit,” and “intentional vs. incidental” are not exactly the same (the first referring mostly to the brain areas associated with conscious vs. unconscious access, the second to the processes involved in the encoding and storage of information based on a single event vs. extended practice, and the third to participants’ passive vs. active orientation toward the encoding and retrieval of the information presented in the task, respectively—see Sawi and Rueckl, 2019 for details), there is a substantial overlap between them. Thus, the terms “incidental-implicit-procedural” and “intentional-explicit-declarative” have been used here, as well as in current SL research, interchangeably (see Conway and Christiansen, 2006; Thiessen, 2017; Soares et al., 2020), even if not necessarily assuming a one-to-one correspondence between them (i.e., a task presented under intentional conditions does not immediately qualify the processes involved in the encoding and storage of the information as explicit nor it would imply the recruitment of brain areas and mechanisms restricted to conscious processing in the medial-temporal lobe).

Moreover, it is also important to point out that we have resorted to the use of an aSL task modeled from Saffran et al. (1996) instead of another implicit learning task (e.g., artificial grammar learning) for several reasons. Firstly, the aSL task allows testing SL skills at a simpler language level of processing (words level), which seems to be particularly appropriate when studying children with language impairments (see also Soares et al., 2018, 2021c; Jiménez et al., 2020 for other arguments justifying why artificial grammar learning tasks were not used). Secondly, recent neuroimaging studies using functional MRI (fMRI) showed that responses to the statistical regularities (TPs) embedded in the input recruit brain areas associated both with procedural and declarative systems, although the reliance on one or another seems also to depend on the type of instructions (implicit vs. explicit) provided to the participants to perform the task (e.g., Karuza et al., 2013; for a review, see Batterink et al., 2019). These features make the aSL an ideal task to test whether children with DLD indeed mobilize the processes and mechanisms associated with declarative learning to compensate for potential procedural deficits, as the PDH claims. Finally, the aSL task has been successfully applied in electrophysiological (ERP) paradigms both with adults (e.g., Abla et al., 2008; François et al., 2014; Batterink et al., 2015a,b; Soares et al., 2020, 2021a, 2022b; Gutiérrez-Domínguez et al., 2022) and young participants (e.g., Bosseler et al., 2016; Mandikal-Vasuki et al., 2017; Choi et al., 2020; Pierce et al., 2021; Soares et al., 2022b,c). This is of special interest because it allows us not only to study the neural underpinnings of speech processing in the brain as exposure to the input stream unfolds with a high time precision, as mentioned above, but also because it allows us to overcome much of the limitations that the use of the 2-AFC post-learning task to test SL entails. Indeed, in a standard aSL experiment, participants are typically tested on their abilities to extract the regularities embedded in the input (TPs) after the exposure phase has occurred by asking them to identify which element of a pair of stimuli (e.g., a three-syllable nonsense word presented during exposure vs. a foil made of the same syllables but never presented together before) resembles most the stream presented before. If performance exceeds the chance level, SL is assumed to have occurred as only the track of the TPs embedded in the input allows a correct “word” discrimination. However, as an increasing number of authors have been pointed out, it is important to consider that a correct “word” discrimination in that task also depends on other cognitive processes (e.g., such as memory and decision making) that might be not fully developed in children of young ages, hence requiring other tasks and techniques to assess SL in a valid and reliable way (see Lammertink et al., 2019; Arnon, 2020; Lukács et al., 2021; Lukics and Lukács, 2021; see also Siegelman et al., 2017 and Soares et al., 2022a for an extended discussion on the limits of the 2-AFC task even with adult participants).

However, despite all these advantages, studies examining the compensatory role of explicit learning mechanisms in the implicit learning deficits in children with DLD by collecting both behavioral and neural data from the same task, presented to the same participants under incidental and intentional conditions, are, to the best of our knowledge, inexistent. We are only aware of a recent brain study conducted by Evans et al. (2022) that have used an aSL task to test procedural learning in adolescents with and without a history of DLD from which behavioral (2-AFC) data were collected, along with behavioral data from the true/false section of the competing language processing (CLPT) task (a task asking participants to judge the veracity of sentences presented in groups of two, three, four, five, and six sentences) to tap declarative knowledge. Participants also performed a semantic congruency task and an auditory lexical decision task as additional indexes of declarative and procedural functioning, respectively, from which behavioral and ERP data were collected. Results showed that adolescents with a history of DLD revealed intact declarative memory, but impaired procedural memory as assessed by CLP and aSL tasks, respectively. Intact lexical-semantic knowledge was observed from the behavioral results of the semantic congruency task, and a less effective lexical-phonological processing was observed from the behavioral results of the auditory lexical decision tasks (as indexed by lower accuracy in words/nonword responses, but an equal sensitivity to high vs. low-frequency words), in adolescents with a history of DLD vs. controls. The neural data revealed that although adolescents with and without a history of DLD showed similar neural responses (i.e., a larger N400 amplitude to incongruent vs. congruent semantic conditions), differences were observed in the location and the time course of the effect. Furthermore, in the auditory lexical decision task, the neural data showed that while adolescents without a history of DLD showed a larger N400 amplitude for low- vs. high-frequency words, as expected, adolescents with a history of DLD did not show any neural signs of such effect. Instead, their neural responses in that ERP component seem to have been modulated by a word imageability and not a word frequency effect. These results were taken by the authors as evidence for the use of a declarative compensatory strategy by adolescents with a history of DLD once they seem to have based their word/nonword responses on their conceptual knowledge rather than on the computation of the phonological patterns of the words used in the auditory lexical decision task. Even though interesting, this interpretation should be taken with caution, as the lexical decision task manipulating the frequency of occurrence of the words might not be the best proxy for the processing of lexical–phonological information (see Quémart and Maillart, 2016 for a study using an auditory lexical decision task but where the phonotactic probability of the non-words was manipulated instead). Moreover, it is also worth noting that Evans et al. (2022) still tested DM and PM functioning by relying on the use of different tasks, and not on the use of the same task manipulating instructions, as we propose in the current work.

Batterink et al. (2015a, see also Batterink, et al., 2015b), in one of the first studies examining the role of implicit and explicit instructions in the context of a typical aSL task to examine the neural underpinnings of the processes recruited to assist SL, collected behavioral (RTs/accuracy) and ERP data while language unimpaired adults performed a speeded target detection task and a 2-AFC task, combined with a remember/know procedure after the exposure phase. Participants were distributed into two learning conditions: in the incidental condition, participants performed the aSL task without any information regarding the task or the stimuli, whereas in the intentional condition learners received explicit training on the six nonsense words embedded in the speech stream previous to the exposure phase. Results from the target detection task showed intentional learners to be faster and to show larger P300 amplitudes to syllables occurring in more predictable than less predictable positions of the triplet, attributable to SL and the greater involvement of controlled and effortful processes. On the 2-AFC task, intentional learners performed more accurately than incidental learners, and their responses were also associated with subjective feelings of stronger recollection, suggesting that the previous knowledge of the nonsense words strengthened participants’ explicit memory and boosted SL function, as expected. Although providing interesting insights, the fact that the authors have collected data only after the exposure phase, along with having adopted a between-subject design in the manipulation of the instructions, raises concerns since recent studies showed a lot of variability in the way individuals respond to SL tasks, particularly when using linguistic materials (see Siegelman et al., 2018; Soares et al., 2022b).

To overcome such flaws, Soares et al. (2020) used a within-subject design in which participants were firstly presented with the implicit version of the aSL task with three-syllable nonsense words drawn from one syllabary, and, subsequently, with an explicit version of an analogous aSL task using three-syllable nonsense words generated from another syllabary to avoid confounds. Note that due to the nature of the task, the order of the tasks was not counterbalanced across participants since once the task has been performed explicitly it cannot be performed implicitly anymore. Moreover, it is also important to point out that the fact that participants have performed first the implicit SL task and subsequently the explicit version of an analogous SL task, might have also contributed to making the second task really explicit, as intended. This issue is particularly important as previous studies showed the effect of explicit instructions on the SL function to be only observed when instructions are specific enough to allow participants to use them while dealing with task requirements (see Arciuli et al., 2014 for a discussion). Moreover, in that work, Soares et al. (2020) have also used complex speech streams entailing not only a higher number of three-syllable nonsense words than in previous works (eight) but, importantly, “words” presenting different levels of predictability (four high-TP “words” and four low-TP “words”) to mimic natural language closely and to further analyze the limits of SL under more uncertain conditions (see Soares et al., 2020 for details). Although results from the 2-AFC tasks showed that performance was neither affected by the conditions under which the tasks were performed (implicit vs. explicit) nor by the predictability of the nonsense words (high vs. low-TP ‘words’), the neural data showed, however, modulations in the N100 and, particularly, in the N400 components, taken as the neural signatures of words’ segmentation in the brain (see Sanders et al., 2002; Cunillera et al., 2006; De Diego Balaguer et al., 2007; Abla et al., 2008; Soares et al., 2020, 2021a, 2022b). The auditory N100 ERP component has been associated with the processing of the sensory features of the stimulus and predictive mechanisms involved in the processing of speech streams (e.g., Heinks-Maldonado et al., 2005). In addition, modulations in the N400 have been proposed to reflect processes related to successful online segmentation of the speech stream into its perceptual units and to the emergence of a pre-lexical trace of words in the brain (see Sanders et al., 2002; Cunillera et al., 2006; De Diego Balaguer et al., 2007; Soares et al., 2020).

Of especial relevance for the purposes of this paper, are the results from a follow-up study (Soares et al., 2022b) in which the authors compared the behavioral and the neural correlates of SL in a group of 5-year-old language unimpaired children to the behavioral and the neural correlates of SL in a group of language unimpaired adults to get new insights into the changes SL might undergo throughout development. Although behavioral (2-AFC) signs of SL were only observed for adult participants, evidence of SL was observed in the N100 and N400 ERP components in both groups, even though a detailed analysis of the neural data revealed some differences between adults and children. For instance, although similar modulations were found in the N100 component in both groups, showing a larger amplitude in the last part relative to the first part of the aSL tasks, differences were observed in the N400 component. In this time window, adults revealed a larger N400 amplitude for the high-TP vs. low-TP “words” regardless of the task, replicating Soares et al. (2020) results, while children showed a more intricate pattern that changed as a function of the predictability of the “words,” especially in the task presented under explicit conditions (a larger N400 amplitude for the low-TP “words” in the first part of the explicit aSL task and for the high-TP “words” in the last part of the explicit aSL task). These findings led the authors to claim that children and adults rely on different mechanisms to assist the extraction of regularities (TPs) embedded in complex speech streams and that SL with auditory linguistic materials is not age-invariant as some authors state (e.g., Reber, 2013; for a review, see Zwart et al., 2017). Anyway, the important point to stress here is that although preschool language unimpaired children failed to reveal behavioral signs of SL, a result that was also observed in other studies with children below 6 years of age (e.g., Raviv and Arnon, 2018; Shufaniya and Arnon, 2018; van Witteloostuijn et al., 2019), the neural results observed in the N400 ERP component showed critically that preschool language unimpaired children were able to take advantage of the previous knowledge of the “word-like” units embedded in the speech streams to boost SL functioning. Thus, the question at stake in the present study is to analyze whether children of the same age with DLD would show a similar pattern of results. Although this is, to the best of our knowledge, the first study conducted in this regard, we hypothesized that if explicit (declarative) learning mechanisms play indeed a compensatory role in implicit (procedural) learning deficits in children with DLD, as the PDH claims, children with DLD should not only present enhanced modulations in the N400 component when the aSL is performed under intentional (explicit) vs. incidental (implicit) conditions, similarly to TLD controls, but also importantly reveal greater differences between the processing of the speech streams under implicit vs. explicit conditions when compared to children from the TLD group. Moreover, differences across the type of “words,” which rely precisely on the computation of syllable TPs, were expected to be lessened in the DLD group due to a strong reliance on explicit (declarative) learning mechanisms to process the speech streams they were exposed to. Although previous studies have failed to show reliable behavioral signs of SL through the use of the offline 2-AFC post-learning task in children below 6 years of age, in this paper we nevertheless opted to collect 2-AFC data from children with and without DLD to further ascertain whether the improvement in SL performance, which children with DLD might reveal when performing the aSL task under explicit conditions, could also be noticed at a behavioral level of analysis.

## Materials and Methods

### Participants

Forty preschool children participated in the study. All were native European Portuguese speakers with normal hearing, as assessed with pure-tone audiometry according to BIAP 02/1 classification (Bureau International Audiofonologie, 2005), and with no neurological or intellectual disabilities. 20 of them, recruited from Speech-Therapist Clinics, presented DLD, while the other 20, recruited from kindergarten institutions, presented typical language development (TLD). Parental informed consent was obtained from all the participants. The study was carried out in accordance with the guidelines of the Declaration of Helsinki and approved by the ethics committee of the local Ethics Committee (University of Minho, SECSH 028/2018).

Children from the DLD and TLD groups were matched on sex, χ^2^(1) = 2.56, *p* = 0.110, age, *t*(38) = 1.36, *p* = 0.183, and in non-verbal IQ, *t*(38) = 1.84, *p* = 0.073 as assessed by the Raven’s Colored Progressive Matrices—Parallel form (CPM-P; Raven et al., 2009). They were also matched in rapid naming both when the time (in seconds), *t*(37) = 1.05, *p* = 0.302, and the number of errors committed producing the name of colors, *t*(37) = 0.86, *p* = 0.396, were taken into account, and in their visuospatial and kinesthetics short-term memory, as assessed by the Corsi block-tapping test, *t*(38) = 0.72, *p* = 0.636, from the Coimbra Neuropsychological Assessment Battery (CNAB; Simões et al., 2016). Children from both groups were also screened on their language abilities by the use of the Preschool Language Assessment (PLA; Mendes et al., 2014), an European Portuguese instrument measuring preschool receptive [listening comprehension (LC)] and expressive [oral verbal expression (OVE)] language skills and metalinguistic awareness (Metalanguage) in the areas of semantics, morphosyntax, and phonology (see Mendes et al., 2014 for details) and through the use of the nonword repetition task of the Language Skills Screening Test (LSST; Viana, 2004) another European Portuguese instrument targeting preschool children. Table 1 presents the demographic, cognitive, and linguistic characteristics of the children included in the DLD and TLD groups in the study.

**Table 1:** Descriptive (Frequencies, Means, and SDs—in brackets) of the characteristics of the children in the developmental language disorder (DLD) and typical language developmental (TLD) groups.

		DLD	TLD
Sex (masculine; feminine)		11; 9	6; 14
Age (years; months)		5;8 (0.43)	5;7 (0.34)
CPM-P scores (percentiles)		62.3 (22.2)	73.9 (17.5)
CNAB scores	Rapid naming (time in s.)	81.7 (28.9)	73.9 (16.5)
	Rapid naming (#errors)	0.68 (1.6)	0.35 (0.8)
	Corsi block-tapping test (%accuracy)	31.9 (11.6)	33.4 (11.9)
PLA scores (percentiles)	Listening comprehension (LC)	56.3(20.9)	74.6 (14.2)
	Oral verbal expression (OVE)	44.3 (22.9)	68.9 (16.9)
	Metalanguage	67.8 (25.0)	90.0 (7.9)
	Total Semantics (LC + OVE)	58.6 (20.2)	73.25 (20.0)
	Total Morphosyntax (LC + OVE)	35.3 (29.5)	73.5 (19.3)
LSST	Nonword repetition (%accuracy)	10.0 (12.6)	32.5 (15.1)

DLD, developmental language disorder group; TLD, typical language development group; CPM-P, Raven’s colored progressive matrices-parallel form; CNAB, coimbra neuropsychological assessment; PLA, preschool language assessment; and LSST, language skills screening test.

As expected, children from both groups differ on their receptive (LC), *t*(38) = 3.22, *p* = 0.003, expressive (OVE), *t*(38) = 3.97, *p* < 0.001, and metalanguage skills, *t*(38) = 3.65, *p* < 0.001, regardless of the language area; as well as in each of the language areas regardless of being receptive or expressive skills, Total semantics, *t*(38) = 2.72, *p* = 0.010, and Total Morphosyntax, *t*(38) = 4.80, *p* < 0.001. Differences were also observed in the nonword repetition task, *t*(38) = 3.67, *p* < 0.001. Taken together, the results obtained from these measures attested the diagnosis of the children in each group and showed that across groups children were also controlled in important demographic and cognitive measures that could impact the results.

### Stimuli

**Figure 1:**
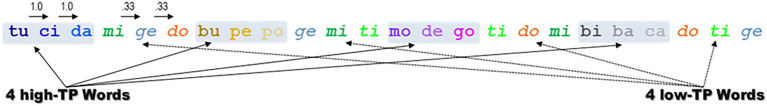
Visual depiction of the high- and low-TP “Words” used in the auditory streams. High-TP, high-transitional probability “words”; Low-TP, low-transitional probability “words.”

The three-syllable nonsense words used in the implicit and explicit versions of the aSL tasks were drawn from Soares et al. (2020). They were made from 32 unique European Portuguese syllables produced and recorded by a native speaker of European Portuguese with duration of 300 ms each. These syllables were distributed into two different syllabaries (A and B) with 16 syllables each to be used in the implicit and the explicit versions of the aSL tasks (counterbalanced across participants). The syllables were concatenated with the Audacity® software (1999–2019) to ensure the absence of any co-articulation cues to affect word segmentation. In each aSL task, four of the nonsense words present TPs between syllables within a “word”of 1.00 (high-TP “words”), whereas the remaining four present TPs within a “word” of 0.33 (low-TP “words”), as in previous works of Soares et al. (2020, 2021a, 2022b). For instance, the nonsense word “*tucida*” presented in Figure 1, which represents a graphic depiction of an auditory stream presented to participants, corresponds to a high-TP “word” as the syllables they entail only appear in that “word” and in that specific syllable positions, while the nonsense word “*migedo*” corresponds to a low-TP “word” as the syllables it entails appear in three different “words” embedded in the stream at different (initial, medial, and final) syllable positions as in the case of the first syllable “*mi*” in the nonsense words “*gemiti*” and “*tidomi*” also presented in Figure 1 (see Soares et al., 2020 for details).

The streams in each of the aSL tasks were edited to contain 60 repetitions of the same nonsense word distributed over six blocks of 10 repetitions each, lasting 1.4 min per block (8.4 min in total). In each block, “words” were presented in pseudo-randomized order to assure that the same “word” or the same syllable will never appear consecutively in a row. In each of the aSL tasks, the stream was also edited to include a randomly superimposed chirp sound (a 0.1 s sawtooth wave sound from 450 to 1,450 Hz) to provide participants with a cover task (i.e., a click detection task), to ensure appropriate attention to the stream as in previous works (see Arciuli and Simpson, 2012; Soares et al., 2020). The target sound was programmed to appear in the interval between syllables in 30% of each “word” type, counterbalanced across syllables to prevent confounds. Correct detections were 130.6 (±8.6) in the implicit aSL task (90.7% of all responses, including false alarms) and 126.8 (±12.7) in the explicit aSL task (88.1%) in children from the DLD group, whereas they were 136.8 (±4.7) in the implicit aSL task (94.9%) and 132.6 (±5.3) in the explicit aSL task (92.2%) in the children from the TLD group. These findings suggest that children from both groups paid appropriate attention to the speech streams in each of the aSL tasks.

The foils used in the 2-AFC tasks were also drawn from the work of Soares et al. (2020). They were made of the same syllables used in the high- and low-TP “words” presented during the exposure phase of each of the aSL tasks, presented with the same frequency and syllable positions to avoid confounds. However, contrary to the syllables in the high- (TP = 1.00) and low-TP “words” (TP = 0.33), the syllables in the foils were never presented together during exposure (TPs = 0). Four lists of materials were created to counterbalance syllables across positions and the type of “words” in each syllabary. Participants in each group were randomly assigned to one list of the Syllabary A and one list of the Syllabary B to perform the aSL tasks either under implicit or explicit conditions. The entire lists of materials are available at https://osf.io/8nx35/?view_only=264c374fa0584584aac85e4b6b39a0b1.

### Procedure

**Figure 2:**
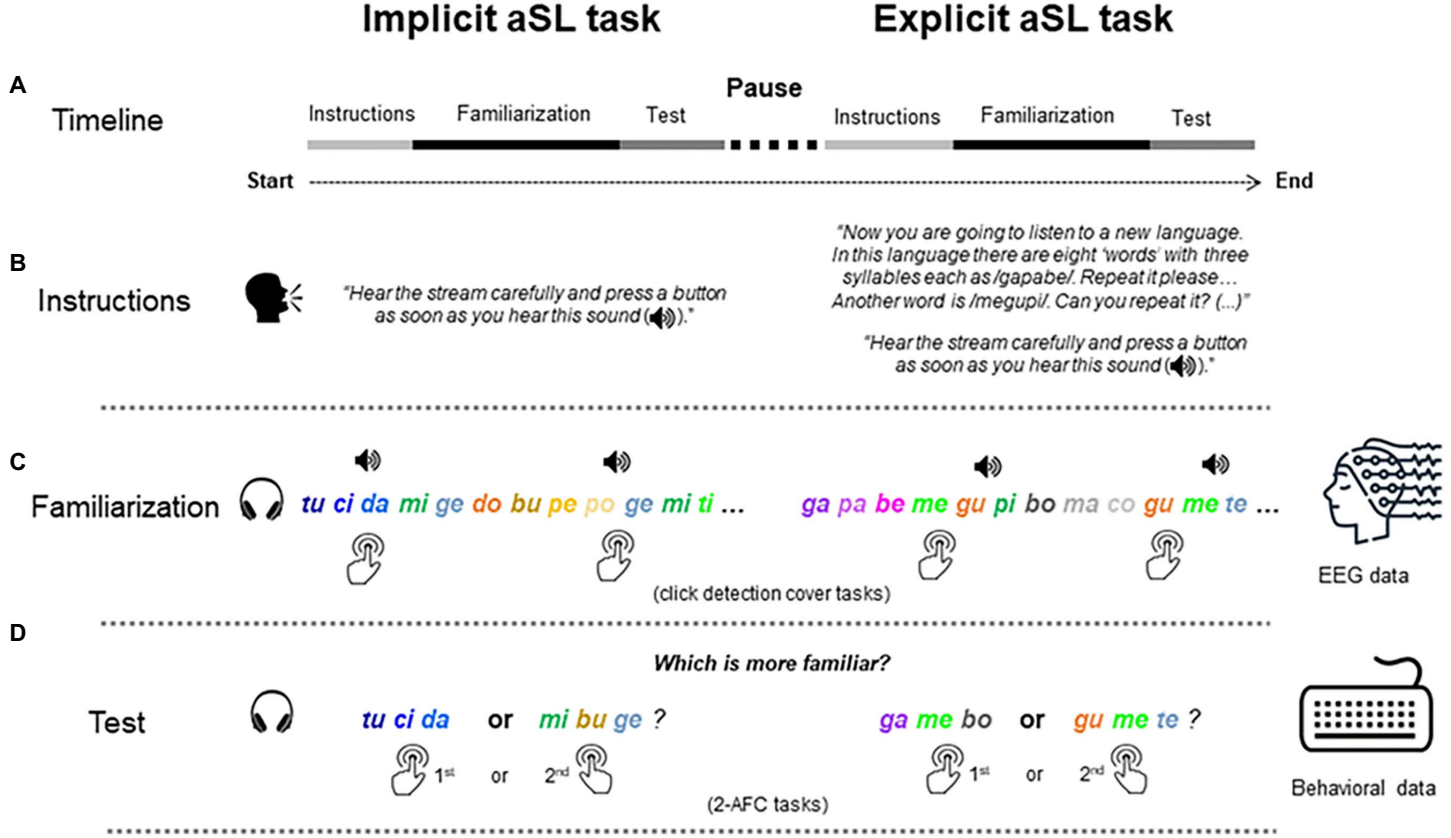
Illustration of the experimental procedure. **(A)** Illustrates the timeline of the experimental procedure in which the implicit and, subsequently, the explicit aSL task were administered. Each aSL task comprised three parts: instructions **(B)**, familiarization **(C)**, and test **(D)** phases. Each task was initiated with specific instructions that determined the conditions under which each of the aSL tasks was performed either without (Implicit aSL) or with the previous knowledge of the task and the structure of the stream used in the experiment (Explicit aSL). In the familiarization or exposure phase of both tasks during which EEG data were collected, participants were presented with a continuous auditory stream of four high-TP and four low-TP “words,” with chirp sounds (depicted as a speaker icon on the figure) superimposed over specific syllables. The chirp sounds could emerge at any of the three syllable positions of the “words,” which precluded its use as a cue for “word” segmentation. During this phase, participants had to perform a chirp detection task. Then, the test phase in each of the aSL tasks consisted of a two-alternative forced-choice (2-AFC) task asking participants to indicate which of the three-syllable sequences (a “word” and a foil) sounded more familiar based on the stream presented during the previous familiarization phase.

EEG data collection was performed in an electric shielded, sound-attenuated room at the facilities of the Psychological Neuroscience Lab, School of Psychology, University of Minho. Participants were seated in a comfortable chair, 1 m away from a computer screen. EEG data were recorded during the exposure phases of each of the aSL tasks with a 64-channel BioSemi Active-Two system (BioSemi, Amsterdam, The Netherlands) according to the international 10–20 system and digitized at a sampling rate of 512 Hz. Electrode impedances were kept below 30 kΩ. EEG was re-referenced offline to the algebraic average of mastoids. Participants were first presented with the implicit version of the aSL task and, subsequently, with the explicit version of an analogous aSL task (see Figure 2). In the implicit version of the task, participants were instructed to pay attention to the auditory stream presented at 60 dB SPL *via* binaural headphones, because occasionally a deviant sound (i.e., a click) would appear, and their task would be to detect it as soon and accurately as possible by pressing the spacebar from the computer keyboard. As mentioned, this functioned as a cover task to assure children paid appropriate attention to the speech streams. Following familiarization, participants were asked to perform the 2-AFC (i.e., to decide which of two auditory stimuli, a “word” and a foil, “sounded more like” the stimuli presented before). The 2-AFC comprised 16 trials (two repetitions of the same nonsense words and foils). Each “word” was paired with two different foils. We opted for this solution instead of presenting each “word” paired exhaustively with each foil (64 trials) as in the work of Soares et al. (2020), because Soares et al. (2022a) recently demonstrated that increasing the number of 2-AFC trials by repeating the same stimuli (“words” and foils) several times throughout the 2-AFC task, worsens SL measurement, as it increases the chances of foils being learned as perceptual units and to interfere with correct “word” discrimination (see Soares et al., 2022a for details).

In the 2-AFC tasks, each trial began with the presentation of a fixation point (cross) for 1,000 ms, after which the first stimulus (“word”/foil) was presented, followed by the second stimulus. A 500-ms inter-stimulus interval separated the presentation of both stimuli. The next trial began as soon as participants made a response or 10 s had elapsed. The 16 trials were presented in two blocks of eight trials each. In each block, the order (first or second) by which the stimuli were presented was controlled for, so that in half of the trials half of the high-TP and half of the low-TP “words” were presented firstly and in the other half the other way around. The trials in each block, as well as the blocks across the task, were randomly presented to the participants. After a brief interval, participants underwent the explicit version of the aSL task. This version followed basically the same procedure adopted in the implicit aSL task, except that previously to the exposure phase participants were presented with additional information about the stimuli that they would listen to during another exposure phase with another set of materials. Specifically, during this training phase, participants were presented auditorily with each of eight new “words” (one by one) and asked to repeat each of them correctly before another “word” was presented. As in the implicit version of the task, during the exposure phase, participants were asked to press a button of the keyword whenever they heard the click sound. After familiarization, participants performed another 2-AFC task similar to the one used previously. The procedure took about 90 min to be completed per participant. Figure 2 presents a visual depiction of the procedure.

## Results

Behavioral (2-AFC) and ERP data analyses were performed using the IBM-SPSS® software (Version 27.0). For behavioral data, the proportion of correct responses was computed for each of the 2-AFC tasks and separately for the high-TP and low-TP “words” in each group of participants (coded as 1 for a correct and 0 for an incorrect response). Grand averages waveforms were calculated in each group for each aSL task and type of “word” separately attending to the length of exposure to the stream (first half vs. second half of each task), to get insights into the temporal dynamics of SL as in previous works. Six participants from the DLD group and four from the TLD group were excluded from the EEG (and also from the behavioral) analyses due to artifact rejection. Data were filtered with a bandpass filter of 0.1–30 Hz (zero phase shift Butterworth). ERP epochs were time-locked to the nonsense words’ onset, from −300 to 1,200 ms (baseline correction from −300 to 0 ms). Independent component analyses (ICA) were performed to remove stereotyped noise (mainly ocular movements and blinks) by subtracting the corresponding components. After that, epochs containing artifacts (i.e., with amplitudes exceeding +/−100 μV) were removed. EEG data processing was conducted with Brain Vision Analyzer, version 2.1.1. (Brain Products, Munich, Germany).

Based on previous literature, mean amplitudes were measured for the following time windows: 80–120 ms (N100 component) and 350–450 ms (N400 component). To account for the topographical distribution of the abovementioned EEG deflections, mean amplitudes’ values were obtained for the topographical regions where amplitudes were maximal: the frontocentral region of interest (ROI; F1, Fz, F2, FC1, FCz, FC2, C1, Cz, and C2) for N100, and the central ROI (FC1, FCz, FC2, C1, Cz, C2, CP1, CPz, and CP2) for the N400. Both for behavioral and ERP data, main or interaction effects that reached statistical or marginal significance levels in comparisons of interest are reported. The Greenhouse–Geisser correction for nonsphericity was used when appropriate. *Post hoc* tests for multiple comparisons were reported after the Bonferroni correction. Measures of effect size (partial Eta squared, η_p_^2^) and observed power (*pw*) for a single effect are reported in combination with the main effects of condition.

### Behavioral Data

The mean proportions of correct responses obtained by each group in the 2-AFC tasks performed under implicit and explicit conditions per type of “word” are presented in Table 2.

**Table 2:** Mean (SD) of the number (Proportion) of correct responses for the High- and Low-TP “Words” in the implicit and explicit auditory SL task (aSL) tasks in the DLD and TLD groups.

Type of “Word” Group	aSL task			
	Implicit		Explicit	
	High-TP	Low-TP	High-TP	Low-TP
DLD	0.51 (0.15)	0.48 (0.15)	0.53 (0.16)	0.53 (0.22)
TLD	0.52 (0.14)	0.44 (0.21)	0.55 (0.17)	0.54 (0.19)

DLD, developmental language disorder group; TLD, typical language development group.

**Figure 3:**
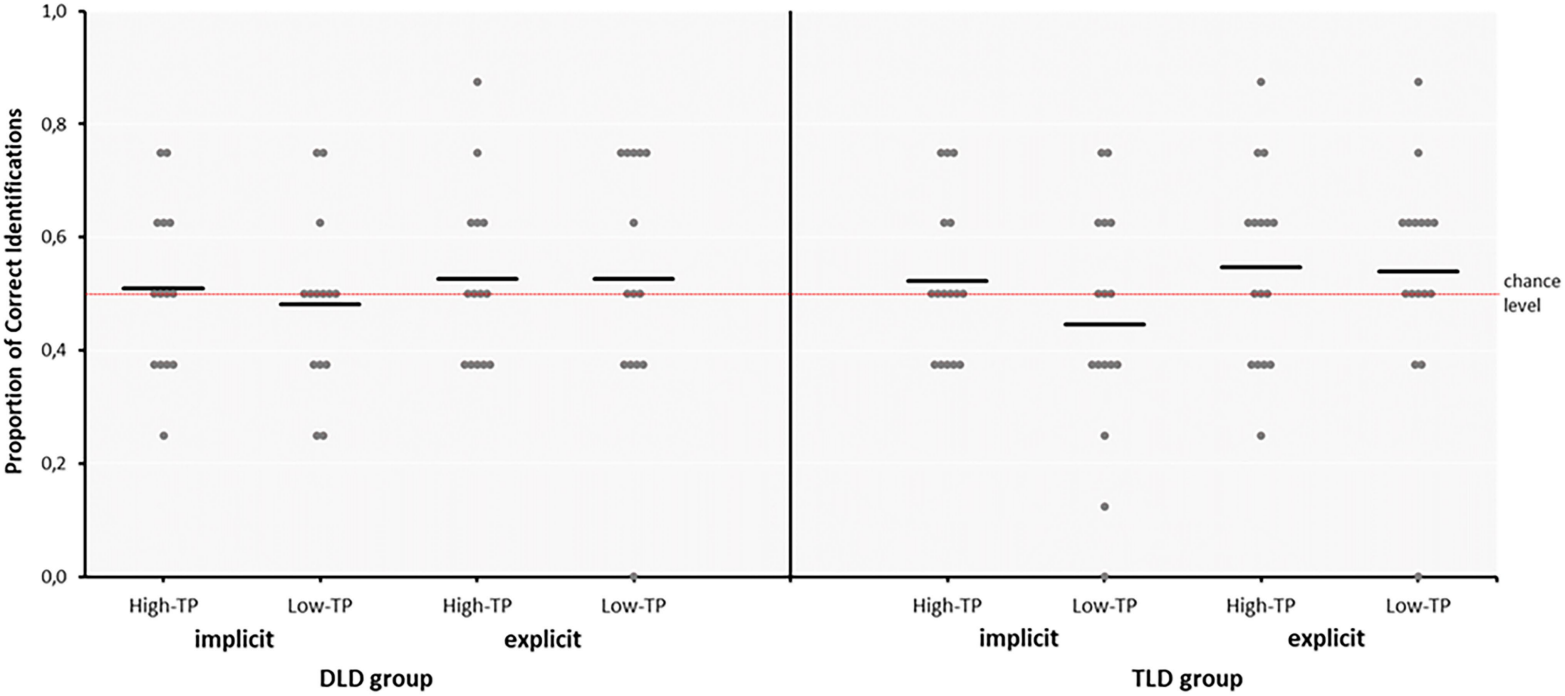
Accuracy Rates (Proportion of Correct Identifications) in the 2-AFC Tasks Performed under Implicit and Explicit Conditions for the high- and low-TP “Words” in the DLD and TLD Groups. DLD, developmental language disorder group; TLD, typical language development group. The dots represent the scores obtained by each participant in each of the conditions (aSL task and type of “word”) per group (DLD and TLD) while the horizontal black solid lines in each of these cases represent the mean of the group in each of these conditions.

As can be seen in Table 2, the results are quite similar across groups and conditions, particularly in the task performed under explicit conditions. In the task performed under implicit conditions, participants from both groups were less accurate at recognizing low- than high-TP “words,” as expected. Nevertheless, the results from the one-sample *t*-tests against chance level failed to reach statistical significance in all the conditions (all *p*s > 0.144), indicating that children from each group as a whole failed to reveal reliable behavioral signs of SL. Nonetheless, the analysis of the individual 2-AFC performance of the children in each group and aSL task showed substantial variability with approximately one-third of children in each group showing a 2-AFC performance above the mean group performance, as depicted in Figure 3.

### ERP Data

**Figure 4:**
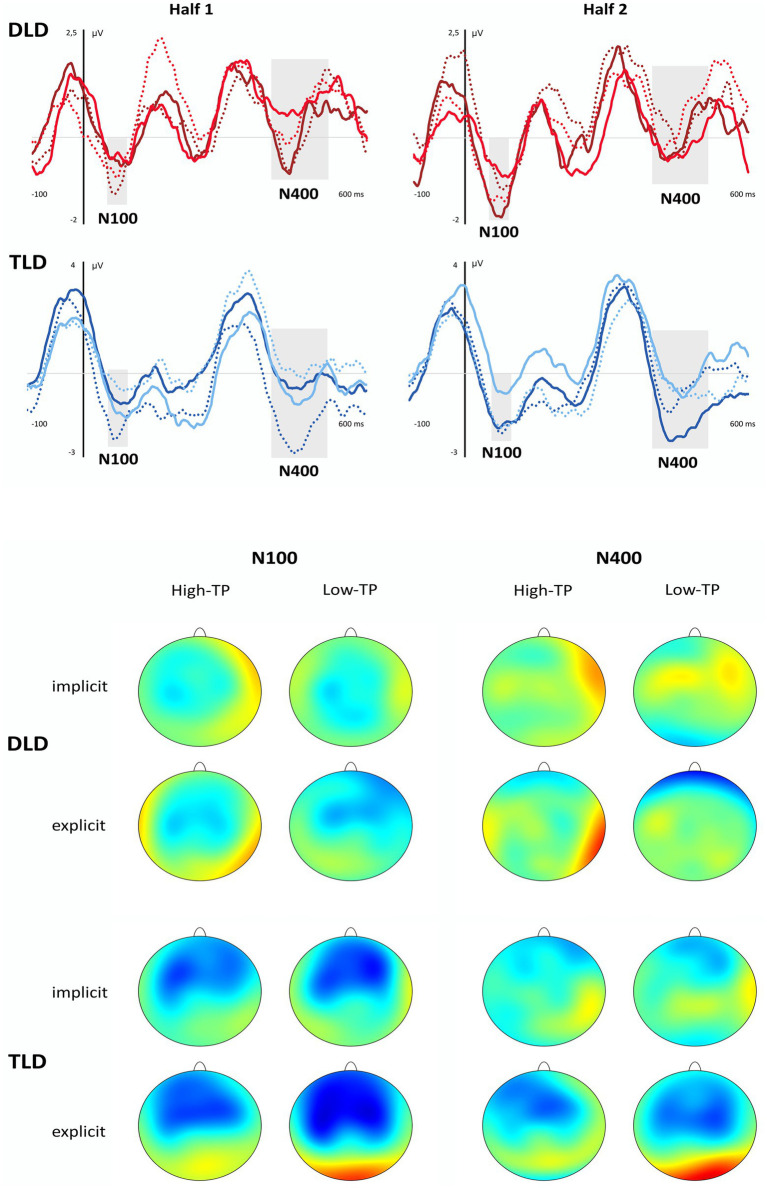
Grand-Averaged Waveforms (Central ROI) and Topographic Maps in the DLD and TLD Groups. In the up panel, the gray shadowed rectangles indicate the analyzed time windows (N100 and N400). DLD, developmental language disorder group: Light red solid line = implicit high-TP condition; Light red dotted line = implicit low-TP condition; Dark red solid line = explicit high-TP condition; Dark red dotted line = explicit low-TP condition. TLD: typical language development group: Light blue solid line = implicit high-TP condition; Light blue dotted line = implicit low-TP condition; Dark blue solid line = explicit high-TP condition; and Dark blue dotted line = explicit low-TP condition. In the bottom panel, values of the topographical images range from −3 to 3 μV in each group and condition.

The up panel of Figure 4 depicts the grand-averaged waveforms (central ROI) obtained from children with and without DLD in the aSL tasks performed under implicit (light lines) and explicit (dark lines) conditions and, in each of them, for the high- (solid lines) and low- (dotted lines) TP “words” in the first half (Half 1) and the second half (Half 2) of each of the aSL tasks. The bottom panel displays the topographic maps obtained in these same conditions.

The results of the ANOVAs conducted for each of the time windows of interest revealed a significant main effect of the length of exposure, maximal at the frontocentral ROI, *F*(1, 28) = 5.80, *p* = 0.023, η_p_^2^ = 0.17, *pw* = 0.64, in the N100 component. This effect indicates that children from both groups showed a larger amplitude in the second half than in the first half of the aSL tasks, regardless of the conditions under which they were performed (implicit vs. explicit) and the type of “words” (high-TP vs. low-TP).

**Figure 5:**
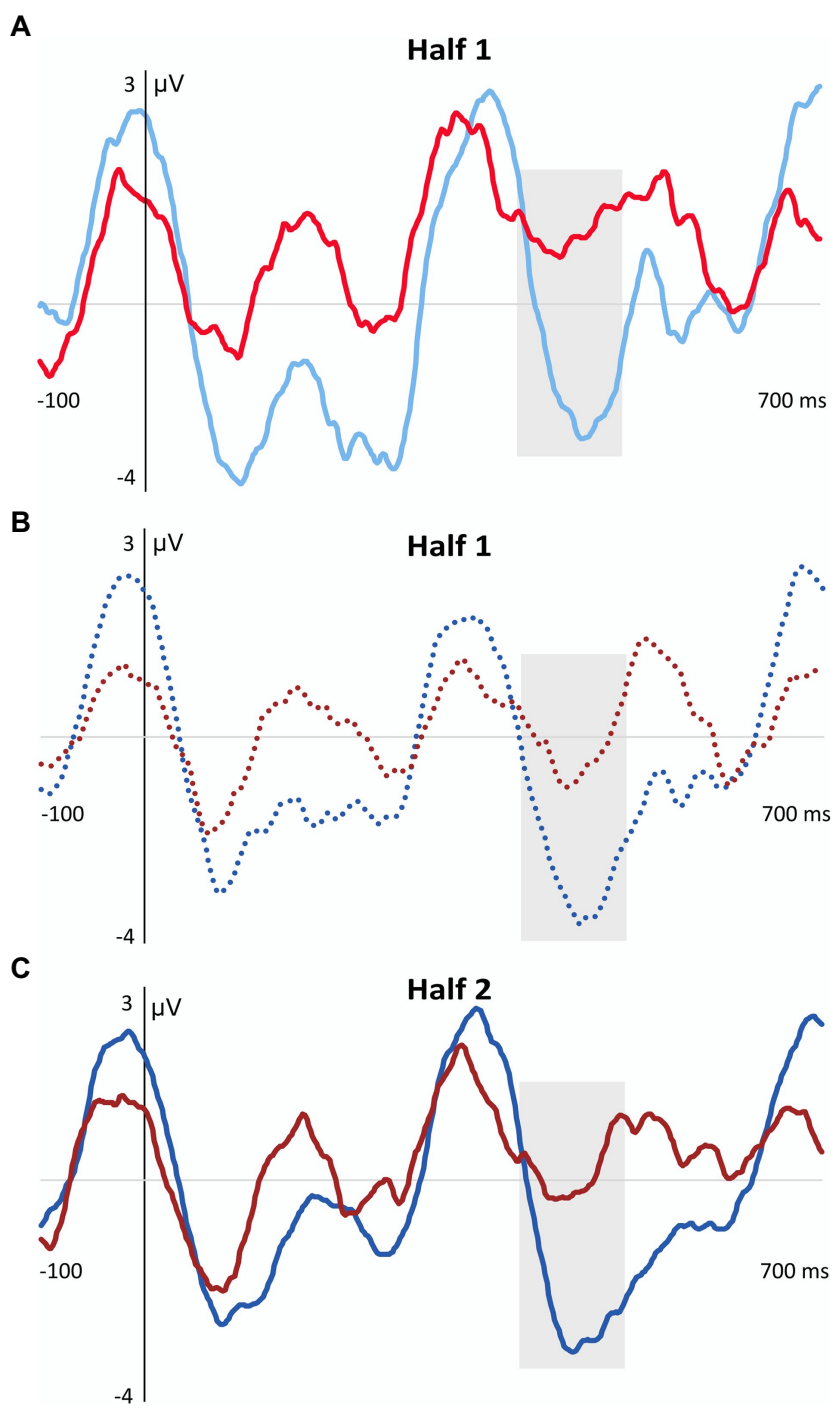
Graphical representation of the Group Effect Observed in the N400 Component for the Implicit and Explicit aSL Tasks as a Function of Type of “Word” and Length of Exposure. Gray shadowed rectangles indicate the N400 time window. **(A)** Group effect in the first half of the implicit aSL task in the high-TP condition. Light red solid line = Development language disorder group; Light blue solid line = Typical language development group. **(B)** Group effect in the first half of the explicit aSL task in the low-TP condition. Dark red dotted line = Development language disorder group; Dark blue dotted line = Typical language development group. **(C)** Group effect in the second half of the aSL explicit task in the high-TP condition. Dark red solid line = Development language disorder group; Dark blue solid line = Typical language development group.

In the N400 component, a significant main effect of task, maximal at the central ROI, was observed, *F*(1,28) = 7.69, *p* = 0.010, η_p_^2^ = 0.35, *pw* = 0.76. This effect revealed that children from both groups as a whole showed a larger amplitude in this component when the task was performed under explicit than implicit conditions, regardless of the type of “words” (high-TP vs. low-TP) and length of exposure to the streams (first half vs. second half). Importantly, the four-way interaction also reached statistical significance in this time window, *F*(1,28) = 5.02, *p* = 0.033, η_p_^2^ = 0.15, *pw* = 0.58. Pairwise comparisons revealed that children from the TLD group showed higher amplitudes than children from the DLD group in the first half of the explicit aSL task for the low-TP “words” (*p* = 0.020), and in the second half of the explicit aSL task for the high-TP “words” (*p* = 0.018). Moreover, a marginal significant group effect was also observed in the first half of the implicit aSL task for the high-TP “words” (*p* = 0.054) indicating a tendency for children from the TLD group to show a larger N400 amplitude than children from the DLD group for the high-TP “words.” Figure 5 depicts a graphical representation of the group effect (DLD group = red lines; TLD group = blue lines) in the aSL tasks performed under implicit and explicit conditions per type of “word” (high-TP = solid lines; low-TP “words” = dotted lines) and length of exposure (first vs. second half).

**Figure 6:**
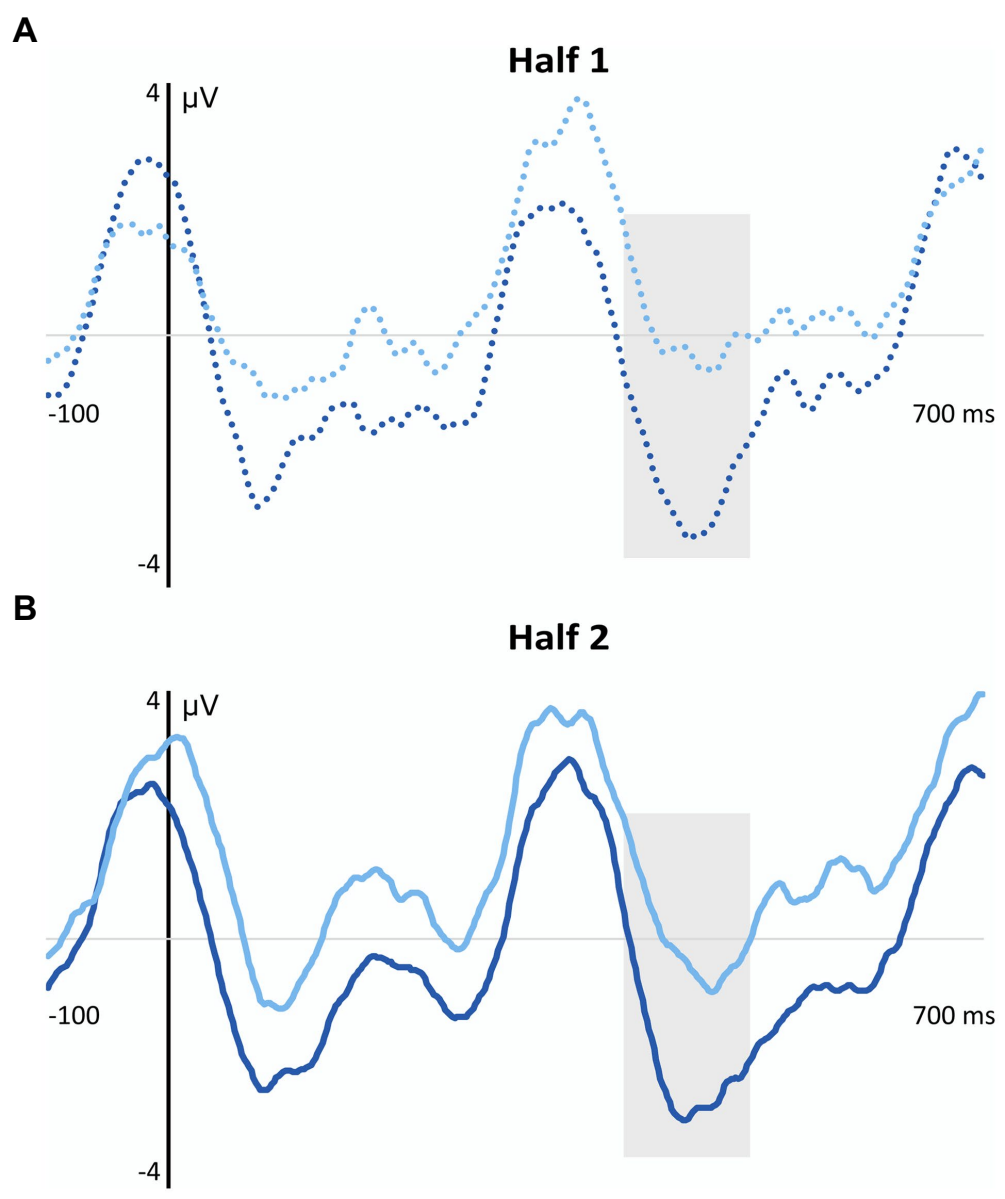
Graphical representation of the aSL Task Effect observed in the N400 Component in the TLD Group as a Function of Type of “Word” and Length of Exposure. Gray shadowed rectangles indicate the N400 time window. **(A)** Task effect in the first half of the explicit aSL task in the TLD group for the low-TP condition. Light blue dotted line = implicit aSL task; Dark blue dotted line = explicit aSL task. **(B)** Task effect in the second half of the explicit aSL in the TLD group for the high-TP condition. Light blue solid line = implicit aSL task; Dark blue solid line = explicit aSL task.

Moreover, the results also revealed that the above-mentioned main effect of task was restricted to children from the TLD group. Indeed, only children from the language unimpaired group showed a larger N400 amplitude in the explicit vs. implicit aSL tasks, even though for the low-TP “words” (*p* = 0.002) in the first half of the explicit aSL task and for the high-TP words (*p* = 0.007) in the second half of the explicit aSL task. Figure 6 depicts the task effects observed in the TLD group (aSL implicit = light blue lines; aSL explicit = dark blue lines) per type of “word” (high-TP = solid lines; low-TP “words” = dotted lines) and length of exposure (first vs. second half).

**Figure 7:**
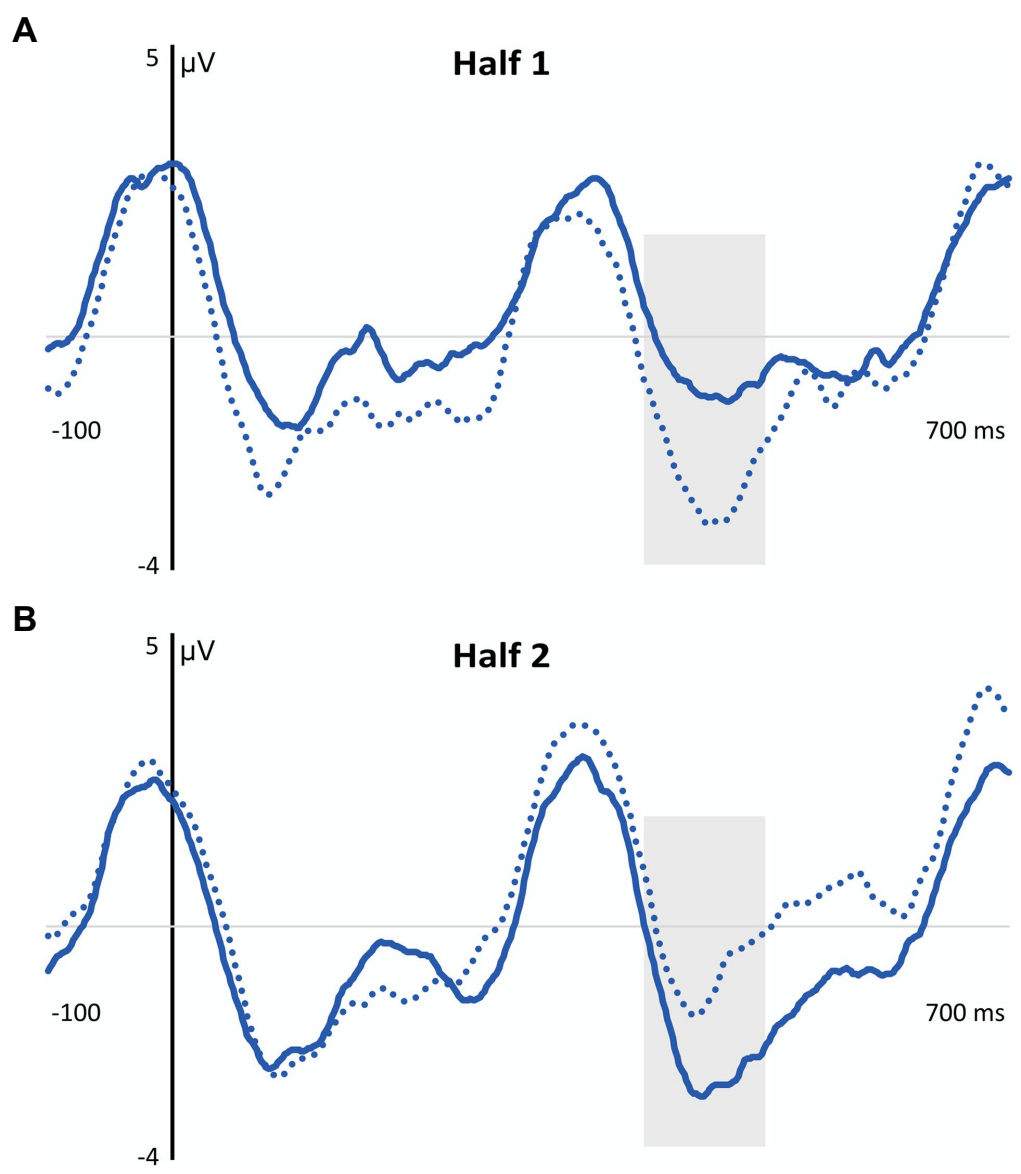
Graphical representation of the Length of Exposure (Panel **A**) and Type of “Word” (Panel **B**) Effects Observed in the N400 Component in the Explicit aSL in the TLD Group. Gray shadowed rectangles indicate the N400 time window. **(A)** Length of exposure effect in the first half of the aSL explicit task in the TLD group. **(B)** “Word” effect in the second half of the explicit aSL task in the DLD group. Dark blue solid line = high-TP condition; Dark blue dotted line = low-TP condition.

In addition, the four-way interaction revealed that children from the TLD group showed a “word” effect in the second half of the explicit aSL task, as reflected in a larger N400 amplitude for the high-vs. low-TP “words” (*p* = 0.044), and also an exposure length effect indicating a larger N400 amplitude in the first half than in the second half of the explicit aSL task for the low-TP “words” (*p* = 0.029). Figure 7 depicts these effects.

## Discussion

The present work aimed to get new insights on the compensatory role that explicit (declarative) learning might play on implicit (procedural) learning deficits in children with DLD, as the PDH claims. For that purpose, we resorted to a new approach that involved the collection of neural (ERP) data, while preschool children, with and without DLD, were exposed to speech streams made of the repetition of four high- and four low-TP three-syllable nonsense words, first under implicit and afterward under explicit conditions—as in Soares and colleagues previous works (Soares et al., 2020, 2021a, 2022b). At the end of the exposure phase of each aSL task, behavioral data were also collected through the use of a standard 2-AFC task. The combination of behavioral and neural measures in a within-subject design allowed us to overcome some of the limitations of previous works specifically those arising from the use of different tasks and stimuli to test PM vs.DM functioning and the exclusive collection of behavioral (RT/accuracy) responses that are strongly affected by attentional and motivational factors, particularly in studies involving language-impaired children from young ages. This is, to the best of our knowledge, the first study using this approach to further analyze the dynamics of DM-PM learning mechanisms in children with DLD (relative to TLD controls), which might provide compelling evidence to ascertain the extent to which explicit learning mechanisms can be effectively mobilized by these children to compensate for implicit learning deficits, as claimed by the PDH with important theoretical and clinical implications.

The results obtained from the 2-AFC tasks showed that children from each group as a whole failed to show reliable signs of SL even though the analyses of their individual performance showed substantial variability in both groups across aSL tasks and type of “words.” The absence of reliable signs of SL even after the “words” have been explicitly taught is not new. Actually, they replicate previous results obtained by our research team with language unimpaired children of the same ages (e.g., Soares et al., 2022b; see also Soares et al., 2021c for similar findings with the artificial grammar learning paradigm), and they also agree with other works using language unimpaired children below 6 years of age (see, for instance, Raviv and Arnon, 2018; Shufaniya and Arnon, 2018 or van Witteloostuijn et al., 2019). Even though it is possible that the absence of behavioral signs of SL in our results may also stem from the complexity of the streams used, which entailed not only a higher number of “words” but “words” more diverse in their internal composition, it is nevertheless important to stress that all those works converge on the view that the 2-AFC task is not well-suited to test SL and that these null results should not be taken as a reflection of “non-learning” but, rather, as the inability of the 2-AFC task to capture SL in children without DLD (Arnon, 2020; Lukics and Lukács, 2021; Soares et al., 2022b).

The ERP data revealed, however, modulations in the N100 and N400 components, taken as the neural signatures of SL in the brain (e.g., Sanders et al., 2002; De Diego Balaguer et al., 2007; Abla et al., 2008; Soares et al., 2020), highlighting, once again, the usefulness of the ERP technique to cope with the limitations of the 2-AFC post-learning tasks to test SL. In particular, the neural results showed enhanced N100 amplitude as exposure to the speech streams unfolded in both groups of participants, regardless of the aSL task and type of “word.” These findings are in line with previous studies and suggest that this component indexes transient effects that change as learning/exposure to the speech streams progresses and the regularities embedded in them are extracted (e.g., Sanders et al., 2002; Cunillera et al., 2006; De Diego Balaguer et al., 2007; Abla et al., 2008; Soares et al., 2022b). They also suggest that the task worked appropriately both for children with and without DLD. Although the absence of reliable behavioral signs of SL might raise some concerns about this interpretation, it is important to note that previous studies conducted with adult participants showed modulations in this component to be associated with the 2-AFC performance. For example, Abla et al. (2008) found that participants who have shown the higher performance in the 2-AFC task showed an increased N100 in the first part of the exposure phase of an aSL task with tones stimuli, while learners with an intermediate 2-AFC performance only showed that N100 enhancement in the last part of the aSL task. In the same vein, Soares et al. (2022b) found evidence for an increased N100 when language unimpaired adults were provided with explicit instructions to perform the aSL task, which also agreed with better 2-AFC performance under explicit conditions. Together, these findings seem to support the view that the increased N100 observed in our data for both groups of participants reflects the recruitment of predictive processes associated with the extraction of regularities embedded in the speech input (Heinks-Maldonado et al., 2005), even if behavioral signs of SL were not observed. Critically, they also showed this brain component to be observed not only in 5-year-old children without language impairments, as previously found by Soares et al. (2022b), but, also in children with DLD, suggesting this neural index of SL to be an early-maturing skill supporting language acquisition, as some authors claim (Saffran et al., 1996; Romberg and Saffran, 2010; Saffran, 2018, 2020) even if less efficiently in children with DLD than TLD controls as the results observed in the N400 component seem to suggest.

Indeed, even though the results observed in that time window, assumed to index processes related to a successful segmentation of the speech stream into perceptual units (word-like) in the brain (e.g., Sanders et al., 2002; Cunillera et al., 2006; De Diego Balaguer et al., 2007; Abla et al., 2008; Soares et al., 2020), indicated that children from both groups showed an enhancement in the N400 component when the task was performed under explicit rather than under implicit conditions, a result also observed in previous works conducted with language unimpaired participants (e.g., Daltrozzo and Conway, 2014; Batterink et al., 2015a,b; Soares et al., 2020, 2021a, 2022b); the four-way interaction observed in this ERP component revealed, however, that only children from the TLD group seem to have taken advantage of the previous knowledge to enhance SL functioning. Note that, within our framework, evidence for a compensatory role of explicit (declarative) learning on implicit (procedural) learning deficits would be indexed not only by enhanced modulations in this ERP component when the “word-like” units embedded in the speech streams were explicitly taught (vs. when they were not), but, importantly, that differences between the processing of the speech streams under implicit vs. explicit conditions would be greater for children from the DLD than for children from the TLD group. However, the results showed the reverse. Indeed, not only the group differences reveal that children from the TLD group showed larger N400 amplitudes than children from the DLD group both in the implicit (even though this effect, observed for the high-TP “words” in the first part of the task, was only marginally significant) and explicit aSL task (for low-TP “words” in the first part of the task and for high-TP “words” in the second half of the task) but, notably, that the differences across tasks only reached a statistically significant level for children from the language unimpaired group. These results agree with other works showing DM deficits in children with DLD (e.g., Lum et al., 2010; Bishop and Hsu, 2015; Kuppuraj et al., 2016; Lukács et al., 2017; McGregor et al., 2017; Lee, 2018; Haebig et al., 2019), thus failing to provide support for the compensatory role of DM in DLD, as the PDH claims (Ullman and Pierpont, 2005; Ullman et al., 2020). They also agree with a recent neuroimaging study using the diffusion tensor imaging (DTI) technique (Lee et al., 2020) showing dysfunctions in the white matter of the brain structures supporting both procedural and declarative functioning in adolescents and young adults with DLD relative to TLD controls.

Nonetheless, before strong conclusions can be drawn, it is also important to consider these results to have arisen from the type of stimuli used in our aSL tasks, once evidence showing DM impairments in children with DLD tends precisely to come from studies using verbal materials, as in our case (e.g., Lum et al., 2010; Bishop and Hsu, 2015; Lukács et al., 2017; McGregor et al., 2017; Haebig et al., 2019). Thus, it is possible to argue these results have stemmed from the difficulties that children from the DLD group present in the encoding and storing of the phonological information of the new “words” rather than from difficulties in using explicit knowledge/explicit learning mechanisms to assist SL *per se* (see Alt and Plante, 2006; Lum et al., 2012, 2015). This possibility should be considered, as children from the DLD group present, indeed, lower phonological working memory skills than children from the TLD group, as assessed by the nonword repetition task from the LSST (see Table 1), and these skills were proven to be strongly related to declarative memory functioning (e.g., Alt and Plante, 2006; Coady and Evans, 2008; Lum et al., 2012, 2015; Arthur et al., 2021). To explore the role that this variable might have played in the results, we conducted yet another analysis based on the same factorial design reported in the Results section but taking the scores obtained in the nonword repetition task into account (i.e., as a covariable in the ANOVAs). Even though the four-way interaction failed to reach statistical significance, due possibly to the lack of statistical power, further exploration of the results revealed nevertheless that the *post hoc* contrasts where the effects tended to reach statistical significance were exactly the same, thus ruling out the phonological working memory skills as the main driving force behind the results. Moreover, it is also important to consider that presenting such complex speech streams during 8.4 min might not suffice to allow children from the DLD group to use the cues embedded in the speech streams and/or the previous knowledge of the “word-like” units in a more efficient manner. For example, Tomblin et al. (1997), in one of the first studies examining PM deficits in adolescents with and without DLD using a serial reaction time task, found that despite adolescents with DLD showed slower learning rates than controls, at the end of the training, performance did not differ between groups. Also, Evans et al. (2009) using an aSL task similar to the one used here but with a lower number of “words” (six) in children with DLD relative to TLD controls, showed that although after 21 min of exposure children from the DLD group performed at chance in the post-learning 2-AFC task, when the time of exposure was doubled performance was significantly greater than chance. Future research should thus test whether extending the time of exposure to the speech streams would make children from the DLD group show a pattern of neural responses similar to the children from the TLD group, which might have important clinical implications. Note that if the same pattern of results emerges, even with extended exposure, this might suggest that using explicit instructions, a strategy that characterizes most of the language interventions in children with DLD (see Ebbels, 2014), might not be well-suited to help DLD children to overcome their language difficulties once they capitalize on skills that might also be impaired in this group of children. Clinical experiments that contrast the effectiveness of language interventions in children with DLD using implicit vs. explicit methods should also be conducted to address this important issue.

Finally, it is also worth mentioning that the results observed here in children from the TLD group replicate Soares et al. (2022b) findings and suggest that, conversely to children from the DLD group, children from the TLD group seem to have taken advantage of the knowledge generated from the previous presentation of the “word-like” units embedded in the speech streams to boost SL functioning. Moreover, they also showed the advantage of the explicit instructions to have affected first the low-TP “words” and only at a later stage the high-TP “words.” This “word” type effect, already observed by Soares et al. (2022b), was accounted by the authors based on two possible explanations: (i) children used the prior knowledge generated from the previous presentation of the “word-like” units to assist the extraction of the most difficult “words”—note that the low-TP “words” are made up of syllables that were also found in other “words” embedded in the stream, which might make these “words” harder to extract and to produce less robust/stable perceptual representations (see Smalle et al., 2016 for evidence of the interference effect generated by item-overlap in a Hebb repetition task); (ii) children relied on syllable frequency instead of syllable TPs to assist word segmentation—note that despite high- and low-TP “words” were presented exactly the same number of times (*N* = 60) during the exposure phase to control for ‘word’ frequency effects (see Soares et al., 2015, 2019), the fact that low-TP “words” involved the encoding of a smaller number of syllables than high-TP “words” (4 vs. 12, respectively) and syllables that occurred three times more frequently than the syllables of the high-TP “words,” might have made children to rely on a simpler strategy to predict the upcoming segment, hence relying on the syllable frequency instead of syllable TPs to create perceptual units beyond the syllable level.

Even though the current work was not designed to disentangle these two proposals, it is nevertheless important to stress that the effect observed in the first part of the implicit aSL task (even if marginal) seems to rule out the second proposal. Indeed, when the aSL task was performed under incidental conditions, children from the TLD group tended to show a larger N400 amplitude for the high-TP “words” in the first part of the implicit aSL task than children from the DLD group, whereas when the aSL task was performed under intentional conditions, children from the TLD group showed a larger N400 amplitude for the low-TP “words” in the first part of the explicit aSL task relative to children from the DLD group.

The result observed in the first part of the implicit aSL task for children from the TLD group suggests that when children performed the task without any information about the task or the stimuli, syllable TPs rather than syllable frequency seems to automatically drive word segmentation. This interesting result suggests that the previous presentation of the “word-like” units embedded in the speech streams might have interfered with the way children usually processed the speech streams to which they were exposed by disrupting a type of processing (based on the extraction of syllable TPs) that might indeed be automatically projected to segment the continuous speech input into word-like units to support language acquisition (Saffran et al., 1996; Romberg and Saffran, 2010; Saffran, 2018, 2020). It is also possible to anticipate that the prior presentation of the word-like units embedded in the speech streams has taxed processing more strongly making children rely on simpler statistics (syllable frequency) to identify the “word-like” units previously presented during exposure. Note that, unlike the implicit aSL task, in the explicit aSL task, children had to simultaneously attend to the “words” previously presented, to the clicks appearing occasionally in the stream, and to the auditory stimuli itself, which was certainly much demanding, justifying the shift in the statistics that children seem to have relied on when the task was performed under implicit vs. explicit conditions, at least when complex speech streams were used. In the same vein, it is possible to anticipate that the capacity limits for information processing that preschool children with DLD typically present in working memory, inhibition, and shifting abilities (see Vissers et al., 2015 for a review), have also hampered the ability of children with DLD to have taken advantage of the previous knowledge of the “word-like” units to boost SL functioning, even if using a simpler strategy as children without language impairments seem to have done. Future research should thus be conducted to analyze if presenting less complex speech streams to children with DLD (made of a lower and/or a less diverse type of “words”) and/or with extended exposure to the speech streams would produce similar results. If future research confirms these results, this would also recommend amendments in the PDH, namely regarding two important assumptions: children with DLD have a spared or even an enhanced DM functioning, and these strengthened DM skills can be used to compensate for their PM deficits in language acquisition. Future research should also test whether similar results would be obtained when using other tasks and paradigms, namely those allowing for the counterbalance of the order of the tasks presented to the children once the nature of the SL task used here made the implicit followed by the explicit presentation of the SL task the only viable solution in this type of design.

## Conclusion

The present study sheds light on the dynamics between implicit–explicit learning mechanisms in children with DLD using a new approach that combined the collection of neural and behavioral data from the same participants (children with DLD and TLD as controls) during the exposure phase of analogous versions of the same aSL task presented under implicit and explicit learning conditions. This new approach allowed us not only to control for differences in the results that might have arisen in previous studies from the use of different tasks and stimuli to test DM and PM functioning but, importantly, to directly examine the changes that performing analogous versions of the same task presented under different learning conditions produced in the SL functioning. This is, to the best of our knowledge, the first study adopting this approach to further examine the compensatory role that explicit learning mechanisms might play on implicit learning deficits in children with DLD, as the PDH claims. Although future studies are required, our findings failed to support the compensatory role of explicit learning mechanisms in the implicit learning deficits in children with DLD, which might have important theoretical and clinical implications.

## Data Availability Statement

The datasets presented in this study can be found in online repositories. The names of the repository/repositories and accession number(s) can be found at: https://osf.io/8nx35/?view_only=264c374fa0584584aac85e4b6b39a0b1.

## Ethics Statement

The studies involving human participants were reviewed and approved by University of Minho, SECSH 028/2018. Written informed consent to participate in this study was provided by the participants’ legal guardian/next of kin.

## Author Contributions

AS conceptualized the study and wrote the first draft of the manuscript. F-JG-D and HO implemented the experiment. AL, NG, and AP collected the data. AS, F-JG-D, HO, DT, and ML analyzed and interpreted the data. F-JG-D, HO, DT, and ML critically revised it. All authors contributed to the article and approved the submitted version.

## Funding

This study was conducted at the Psychology Research Center (PSI/01662), University of Minho, and supported by the Grant POCI-01-0145-FEDER-028212 from the Portuguese Foundation for Science and Technology and the Portuguese Ministry of Science, Technology and Higher Education through national funds, and co-financed by FEDER through COMPETE2020 under the PT2020 Partnership Agreement and within CINTESIS, R&D Unit (references UIDB/4255/2020 and UIDP/4255/2020).

## Conflict of Interest

The authors declare that the research was conducted in the absence of any commercial or financial relationships that could be construed as a potential conflict of interest.

The reviewer AV declared a shared affiliation with the author ML to the handling editor at the time of review.

## Publisher’s Note

All claims expressed in this article are solely those of the authors and do not necessarily represent those of their affiliated organizations, or those of the publisher, the editors and the reviewers. Any product that may be evaluated in this article, or claim that may be made by its manufacturer, is not guaranteed or endorsed by the publisher.
